# An overview of Internal Medicine Point-of-Care Ultrasound rotations in Canada

**DOI:** 10.1186/s13089-022-00287-1

**Published:** 2022-09-02

**Authors:** Mathilde Gaudreau-Simard, Katie Wiskar, Elaine Kilabuk, Michael H. Walsh, Michael Sattin, Jonathan Wong, Zain Burhani, Shane Arishenkoff, Jeffrey Yu, Ada W. Lam, Irene W. Y. Ma

**Affiliations:** 1 Division of General Internal Medicine, Department of Medicine, The Ottawa Hospital, University of Ottawa, 1053 Carling Ave, D 107, Box 209, Ottawa, ON K1Y 4E9 Canada; 2 Division of General Internal Medicine, Department of Medicine, University of British Columbia, Vancouver, BC Canada; 3 Division of General Internal Medicine, Department of Medicine, University of Ottawa, Ottawa, ON Canada; 4 Division of General Internal Medicine, Department of Medicine, University of Calgary, Calgary, AB Canada; 5 Division of General Internal Medicine, Department of Medicine, Western University, London, ON Canada; 6 Division of General Internal Medicine, Department of Medicine, University of Alberta, Edmonton, AB Canada; 7 Division of Emergency Ultrasound, Department of Emergency Medicine, Massachusetts General Hospital, Boston, MA USA; 8 Department of Community Health Sciences, University of Calgary, Calgary, AB Canada

**Keywords:** Point-of-care ultrasound, POCUS, Internal Medicine, Education

## Abstract

**Background:**

Point-of-care ultrasound (POCUS) is a growing part of internal medicine training programs. Dedicated POCUS rotations are emerging as a particularly effective tool in POCUS training, allowing for longitudinal learning and emphasizing both psychomotor skills and the nuances of clinical integration. In this descriptive paper, we set out to review the state of POCUS rotations in Canadian Internal Medicine training programs.

**Results:**

We identify five programs currently offering a POCUS rotation. These rotations are offered over two to thirteen blocks each year, run over one to four weeks and support one to four learners. Across all programs, these rotations are set up as a consultative service that offers POCUS consultation to general internal medicine inpatients, with some extension of scope to the hospitalist service or surgical subspecialties. The funding model for the preceptors of these rotations is predominantly fee-for-service using consultation codes, in addition to concomitant clinical work to supplement income. All but one program has access to hospital-based archiving of POCUS exams. Preceptors dedicate ten to fifty hours to the rotation each week and ensure that all trainee exams are reviewed and documented in the patient’s medical records in the form of a consultation note. Two of the five programs also support a POCUS fellowship. Only two out of five programs have established learner policies. All programs rely on In-Training Evaluation Reports to provide trainee feedback on their performance during the rotation.

**Conclusions:**

We describe the different elements of the POCUS rotations currently offered in Canadian Internal Medicine training programs. We share some lessons learned around the elements necessary for a sustainable rotation that meets high educational standards. We also identify areas for future growth, which include the expansion of learner policies, as well as the evolution of trainee assessment in the era of competency-based medical education. Our results will help educators that are endeavoring setting up POCUS rotations achieve success.

## Background

Point-of-care ultrasound (POCUS) is increasingly recognized as an invaluable tool for the care of internal medicine (IM) patients [1–3]. As a result, there is a growing need to incorporate POCUS into Canadian IM residency training programs [4–7]. A multifaceted approach is required to build an effective POCUS educational program, incorporating elements such as didactic teaching, hands-on training with standardized patients, and simulation or task trainer usage [8, 9]. In addition, a crucial element of acquiring POCUS competence is longitudinal, spaced learning [10, 11], where integrated training and learning occurs over time. This element is educationally important as workshops and didactic lectures alone often fail to confer POCUS competence. In light of evidence-based educational principles, POCUS rotations are emerging as a particularly effective training modality, as they offer learners dedicated time to acquire both the psychomotor and cognitive skills required to perform POCUS in the clinical setting, while allowing for deliberate practice [9, 12].

As POCUS rotations become more widespread in IM training, we set out to review the current state of IM POCUS rotations in Canada. To our knowledge, there are currently five Canadian IM training programs that offer a POCUS rotation. We present an overview of these rotations and highlight key elements that are required for a sustainable, high value educational experience. Our goal is to share our lessons learned with educators who are considering setting up a dedicated IM POCUS rotation at their center.

## Methods

POCUS leaders at each of the 17 IM residency training programs across Canada were contacted to identify programs that offer an IM POCUS rotation. We received a response from all seventeen  programs, and five were identified as offering an IM POCUS rotation for the 2021–2022 academic year. These included the University of Alberta (“Alberta”), the University of British Columbia (“British Columbia”), the University of Calgary (“Calgary”), the University of Ottawa (“Ottawa”), and Western University (“Western”). Subsequently, POCUS leads from each of these programs were invited to describe their local environment via a short online survey and to co-author this paper.

## Results

### General characteristics

Table 1 presents general characteristics for the five Canadian IM POCUS rotations.

**Table 1:** Characteristics of 2021–2022 internal medicine point-of-care ultrasound rotations

		University of Alberta	University of British Columbia	University of Calgary		University of Ottawa	Western University
				FMC	RGH		
Inception date (academic year)		2020–2021	2017–2018	2017–2018	2021–2022	2021–2022	2017–2018
Length of rotation (weeks)		2	4	4	4	4	4
# of blocks offered/year		13	13	6	13	2	9
# of learners/block		1	2–4	2–4	1–4	2	2–3
# of IM POCUS attendings		12^1^	4	7		2	3
# of POCUS fellowship trained attendings		1	5	7		1	2
# of IM POCUS fellows		0	1–2	2	0	0	0
Estimated # of exams performed per week	Diagnostic^2^	1–2	10–20	25–30	20–25	8–16	18–22
	Procedural^3^	5–10	10–15	10–15	5–10	2–3	0

*FMC* Foothills Medical Center, *RGH* Rockyview General Hospital, *IM* Internal Medicine

^1^This reflects the number of attendings who supervise the rotation and who are trained in ultrasound-guided procedures. Three of these are POCUS-trained with a broader scope of skills, including diagnostic applications of POCUS

^2^The  three most common indications across all sites are undifferentiated hypoxia, undifferentiated shock, and volume status assessment. Other common indications are chest pain, sepsis, acute kidney injury, deep vein thrombosis, joint effusions, abscess, and assessment for ascites

^3^The two most common procedures across all sites are paracentesis and thoracentesis. Other common procedures are vascular access, lumbar puncture, and arthrocentesis

### Structure and scope

All programs structure their POCUS rotation as a consultative service that runs from 9 am to 5 pm, Monday to Friday. A POCUS attending is assigned to the service each week, except for one site where attendings rotate more frequently (Calgary). At four of the five programs (British Columbia, Calgary, Ottawa, and Western), the POCUS service predominantly accepts consultation requests from the clinical teaching units (CTU) or internal medicine consultation services. Three of these also receive occasional requests from the hospitalist service, palliative care, or surgical specialties (British Columbia, Calgary, and Western). One of the programs (Alberta) offers a broader hospital-wide POCUS consultation service including surgical services, internal medicine subspecialties, and family/hospitalist medicine. In three of the five programs (British Columbia, Calgary, and Ottawa) the service offers a mix of diagnostic and procedural POCUS. One of the programs (Western) offers exclusively diagnostic POCUS consultations and one program (Alberta) offers predominantly procedural consultations with rare diagnostic POCUS consultations (estimated at 10% of their activity).

### Workflow

All programs have adopted a similar workflow. Exams are either performed under direct supervision or performed independently by the trainee and reviewed asynchronously by the POCUS attending or fellow. The ratio of exams that are performed under direct supervision to those that are reviewed asynchronously varies greatly and is dependent upon the number of exam requests and the concomitant clinical demands of the POCUS supervisor.

Exams that are performed independently by trainees are saved either via hospital-based archiving or an online platform that allows sharing of exams if archiving is not available (British Columbia). Once the exam has been reviewed by the POCUS attending or fellow, findings are documented in the patient’s chart in the form of a consultation note that includes POCUS results and associated clinical recommendations. Across all programs, exams are reviewed by a POCUS attending or fellow before issuing a clinical recommendation to the most responsible physician (MRP) team. Considering that attendings may have concomitant clinical demands, reviewing exams may be delayed to the end of the day. However, trainees are encouraged to notify their POCUS supervisors if a critical finding is identified or if a consult is urgent, so that the POCUS supervisor can prioritize accordingly.

Across all programs, diagnostic exams performed outside of a consultation request (i.e., purely educational scans) are very rare (estimated at < 1% of the service’s activity in Alberta, British Columbia, Calgary, and Western and < 10% in Ottawa). In the rare event that these are performed, permission is sought from the MRP team, informed consent is obtained from the patient, and the exam follows the same reviewing process as clinical exams. Detailed documentation in the format of a consultation note is not pursued.

### Resources and requirements

Table 2 presents an overview of the POCUS resources available at each site and requirements for each rotation, if present.

**Table 2:** Key features of 2021-2022 internal medicine POCUS rotations

	University of Alberta	University of British Columbia	University of Calgary		University of Ottawa	Western University
			FMC	RGH		
Resources						
*How many dedicated internal medicine ultrasound machines are available at each site offering an IM POCUS rotation *[13]*?*						
Hand-held	1	2	1	1	1	1
Cart-based	1	4	4	2	2	5
*What is your funding model for the preceptors on the rotation?*						
Fee-for-service	Yes	Yes	Yes	Yes	Yes	Yes
Salaried	No	No	Yes	No	No	No
Stipend	No	No	No	No	No	No
Concomitant clinical work	Sometimes Clinics, CTU, consults	Sometimes CTU, consults	Sometimes CTU, consults	Always Clinics	Usually Consults	Sometimes Clinic, CTU, consults,
*How many hours per week would you estimate the dedicated POCUS attendings devote to the POCUS service (didactic and hands-on teaching as well as reviewing exams)?*						
	10–20	25–35	30–40	25	25–30	30–50
*Does your program have access to hospital-based archiving?*						
	Yes	No (in process)	Yes		Yes	Yes
*Does your program offer an IM POCUS fellowship?*						
	No	Yes	Yes		No	No
Requirements						
*Are there prerequisites for trainees wishing to participate in the rotation?*						
	PGY4/5 only	PGY2 or above	PGY 2 or above	PGY 2 or above	PGY4/5 only	PGY2 or above
*Is there an exit evaluation for trainees who complete the rotation?*						
	Across all sites, trainees are evaluated via an In-Training Evaluation Report (ITER) in addition to Internal Medicine Entrustable Professional Activity for procedural skills					
*Is there a target number of exams to be performed per trainee?*						
*Target # exams*	5 Cardiac 20 Thoracic 10 Abdominal 10 IVC	No set targets	50 Thoracic 5 IJ 10 Inferior epigastric vessels 5 Intercostal vessels 30 IVC 50 FAST^1^	No set targets	25 Cardiac 25 Thoracic 10 Abdominal 10 IVC 5 MSK	30 Cardiac with IVC 20 Thoracic 10 Abdominal
*Does your program have learner policies in place *[13]*?*						
	Yes	No	Yes		No (in process)	No

*CTU* clinical teaching unit, *PGY* post-graduate year, *IJ* internal jugular, *IVC* inferior vena cava, *MSK* musculoskeletal

^1^If Canadian POCUS exam candidate, then also 50 cardiac exams

## Discussion: lessons learned

This comparative exercise has allowed us to identify fundamental elements that should be considered by those seeking to deliver a sustainable, educationally rich POCUS rotation.

### Proposed model: the consultative service

All five programs structure their rotation as a consultative service, providing not only imaging findings, but also integrating findings into clinical recommendations. We see three main advantages to this model. First, it ensures that trainees acquire not only the skills of image acquisition and interpretation, but also learn the more complex skill of clinical integration. Clinical integration requires an understanding of test characteristics, indications, limitations, and scope of POCUS [14]; additionally, it requires the ability to incorporate POCUS findings into the broader picture of the complex medical inpatient with multiple comorbidities. This skill is essential for trainees to safely incorporate POCUS into their practice. Second, a consultative service allows for supervised training in POCUS-guided procedures, which, despite being a core competency for IM residents in Canada [15] is frequently difficult to obtain through existing clinical rotations [5, 7]. Third, an IM POCUS consultative service allows broader access to POCUS for IM patients, which has the potential to improve patient care. For these reasons, we recommend programs looking to offer a POCUS rotation to consider structuring it as a consultative service.

### Inputs and resources to consider

The program planner must account for several important inputs when considering the feasibility of a POCUS rotation. These include human resources, access to archiving, and funding.

### Human resources

Offering a quality POCUS rotation requires a highly motivated, highly trained POCUS workforce. All sites have at minimum one POCUS fellowship-trained internist and a minimum of two POCUS-capable attendings. Given the hands-on nature of the service, a strong preceptor presence is essential and current preceptors dedicate 25–50 hours per week to the service. Careful consideration should be given to available human resources when deciding whether a POCUS rotation is feasible. Depending on the number of POCUS-trained preceptors available, it may be prudent to only offer the rotation for a limited number of blocks to ensure adequate supervision.

As previously alluded to, two programs surveyed (British Columbia and Calgary) have GIM POCUS fellowship programs in place. The experienced POCUS fellow provides additional oversight and teaching for residents which significantly bolsters the ability to run a continuous, robust POCUS service. However, running a high-level POCUS fellowship has numerous additional demands beyond simply running a POCUS rotation, the details of which have been previously described [14].

### Archiving

Although hospital-based archiving is not essential, as proven by the success of one of the programs that currently lacks this resource (British Columbia), we highly recommend that programs looking to set up a POCUS rotation invest in an archiving platform. Archiving ensures that exams are saved in a patients’ medical records, allows remote reviewing of exams and allows for ongoing quality assurance [14, 16]. Echoing our colleagues in Emergency Medicine [17], we believe that archiving should be the standard of care as POCUS becomes more widespread in IM.

### Funding

Lastly, adequate funding is critical for the sustainability of these rotations. Funding models vary across programs, but the majority rely on fee-for-service billings using consultation and procedural codes. Billing rules differ between the provincial insurance plans such that remuneration varies across the programs. Three of the programs have funded positions for POCUS-related academic activities (British Columbia, Calgary, and Western). However, the deliverables attached to this protected time include broader IM POCUS teaching activities, administrative tasks, and research activities rather than the POCUS rotation itself. None of the programs have protected academic time or a stipend dedicated to the rotation itself.

Considering this, as well as the variable remuneration from POCUS consultations alone, attendings across all programs take on various concomitant clinical service to supplement income (see Table 2). Considering the time commitment of POCUS rotations, concomitant clinical duties must be balanced with the need to meet high educational standards for the rotation.

### Education delivery

There is some heterogeneity among the programs surveyed with regard to educational delivery during the POCUS rotation. All programs incorporate significant bedside teaching, which, due to the hands-on nature of the skills involved, is essential for trainee development. Although consensus recommendations for IM POCUS curricula do exist [4], most programs did not have a predictable formal teaching structure during the rotation; rather, teaching is often dependent on attending availability, service needs, and opportune patient presentations. Most programs rely on a combination of in-person didactic lectures as well as self-directed learning and flipped-classroom approaches. Additionally, most programs offer broader POCUS education by requiring that trainees rotating on the POCUS service deliver supervised teaching sessions (academic half day, rounds, bedside scanning) to IM residents on CTU.

### Gaps and areas for improvement

This process allowed us to identify several limitations. First and foremost, we identified that POCUS rotations are offered in only a third of Canadian IM programs. In addition, the programs that do offer a POCUS rotation can only accommodate a handful of trainees. This capacity limitation is driven by the scarcity of POCUS-trained faculty. This means that many IM trainees do not have access to this learning experience and reiterates the importance of broader IM POCUS educational activities beyond a POCUS rotation.

This process also allowed us to identify quality gaps and areas for improvement for those programs that do offer a POCUS rotation, specifically around policies and trainee assessment. Only two programs (Alberta and Calgary) currently have learner policies in place. Other policies such as incidental finding policies are important for patient safety and should be more widely adopted in IM POCUS. Regarding trainee assessment, all programs currently use an In-Training Evaluation Report (ITER) in addition to Entrustable Professional Activities (EPAs) for ultrasound-guided procedures. Though most programs have established a target number of exams, these serve as guidelines for what is minimally achievable over a two to four weeks rotation, rather than a requirement for successful completion of the rotation or achievement of competency. We recognize that there are inherent disadvantages to a numbers-based approach, and portfolio-building is ultimately tailored to learners’ needs [11]. In the era of Competency Based Medical Education (CBME), longitudinal evaluation of competency is the preferred approach [15]. This implies that assessment of competence should occur beyond a two or four weeks rotation and occur throughout training. In this regard, we can learn from our colleagues in Emergency Medicine [18] and Critical Care Medicine [19] who have established POCUS EPAs, allowing for spaced observation of competence throughout training. IM is only starting to plan for the eventual integration of diagnostic POCUS into IM CBME. As evidenced by this review, there is significant heterogeneity in POCUS training across programs and one can infer that trainee skills are equally varied. Ongoing work is required to define IM POCUS competency which will invariably lead to better harmonization of training and skills among IM residents.

## Conclusion

This overview of the five IM POCUS rotations currently offered in Canada highlights common themes and characteristics across the various programs, as well as ongoing limitations and struggles with program development and expansion.

First, providing POCUS as a consultative service not only allows for emphasis on clinical integration of diagnostic POCUS, but also provides proper supervision of POCUS-guided procedures while expanding the reach of POCUS to more patients. Second, operating a POCUS service requires an expertly trained, highly motivated workforce to ensure a quality educational experience for learners. Heavy preceptor involvement is needed to provide didactic teaching, hands-on supervision and reviewing of exams. The latter is facilitated by a hospital-based archiving system, which we recommend for any program considering a POCUS rotation. Third, despite increased uptake and consults for the POCUS service, revenue from POCUS remains overall low and thus most fee-for-service POCUS attendings continue to rely on concomitant clinical work to supplement revenue generated on the POCUS service.

We identified three main weaknesses of the current POCUS rotations: limited capacity, the lack of policies to address quality and patient safety, and the lack of uniform trainee assessments for the diagnostic applications of POCUS. These areas of weaknesses should form the focus of future scholarly work as IM POCUS rotations continue to expand and mature towards educational excellence. Future work should also focus on evaluating the effectiveness of these rotations on learners’ knowledge, skills, and clinical integration of POCUS as well as the impact of an IM POCUS consultation service on resource utilization and patient outcomes.

## Data Availability

Data sharing is not applicable to this article as no datasets were generated or analyzed during the current study.
