# Correction: An overview of internal medicine point-of-care ultrasound rotations in Canada

**DOI:** 10.1186/s13089-022-00288-0

**Published:** 2022-09-14

**Authors:** Mathilde Gaudreau-Simard, Katie Wiskar, Elaine Kilabuk, Michael H. Walsh, Michael Sattin, Jonathan Wong, Zain Burhani, Shane Arishenkof, Jefrey Yu, Ada W. Lam, Irene W. Y. Ma

**Affiliations:** 1 Division of General Internal Medicine, Department of Medicine, The Ottawa Hospital, University of Ottawa, 1053 Carling Ave, D 107, Box 209, Ottawa, ON K1Y 4E9 Canada; 2 Division of General Internal Medicine, Department of Medicine, University of British Columbia, Vancouver, BC Canada; 3 Division of General Internal Medicine, Department of Medicine, University of Ottawa, Ottawa, ON Canada; 4 Division of General Internal Medicine, Department of Medicine, University of Calgary, Calgary, AB Canada; 5 Division of General Internal Medicine, Department of Medicine, Western University, London, ON Canada; 6 Division of General Internal Medicine, Department of Medicine, University of Alberta, Edmonton, AB Canada; 7 Division of Emergency Ultrasound, Department of Emergency Medicine, Massachusetts General Hospital, Boston, MA USA; 8 Department of Community Health Sciences, University of Calgary, Calgary, AB Canada

## Correction: The Ultrasound Journal (2022) 14:37 10.1186/s13089-022-00287-1

Following the publication of the original article [1], we were notified that the first author is only affiliated to Division of General Internal Medicine, Department of Medicine, The Ottawa Hospital, University of Ottawa (affiliation number 1), and not with 7 and 8.

The original article has been corrected.
